# Progress Toward Measles and Rubella Elimination — Indonesia, 2013–2022

**DOI:** 10.15585/mmwr.mm7242a2

**Published:** 2023-10-20

**Authors:** Stephen Chacko, Mushtofa Kamal, Endang Budi Hastuti, Fristika Mildya, Cornelia Kelyombar, Vivi Voronika, Epid, Prima Yosephine, Gertrudis Tandy, Devi Anisiska, Sherli Karolina, Lulu Ariyantheny Dewi, Sudhir Khanal, Sunil Bahl, Fetty Wijayanti, Rebecca D. Merrill, Christopher H. Hsu, Michelle Morales

**Affiliations:** ^1^World Health Organization Country Office for Indonesia, Jakarta, Indonesia; ^2^Ministry of Health Indonesia; ^3^Immunizations and Vaccines Development, World Health Organization South-East Asia Regional Office, New Delhi, India; ^4^Global Health Center, CDC Country Office Indonesia, Jakarta, Indonesia; ^5^Global Immunization Division, Center for Global Health Center, CDC.

**Summary**

**What is already known about this topic?**

In 2019, Indonesia and the other countries in the World Health Organization South-East Asia Region adopted the goal of measles and rubella elimination by 2023.

**What is added by this report?**

During 2013–2021, measles and rubella incidence declined by 95% and 89%, respectively. However, in 2022, measles and rubella incidence significantly increased compared with 2021 because of disruption in surveillance and immunization services caused by the COVID-19 pandemic, leading to gaps in immunity.

**What are the implications for public health practice?**

Indonesia has made substantial progress toward measles and rubella elimination. To achieve elimination, urgent efforts are needed to restore immunization services adversely affected by the COVID-19 pandemic, close immunity gaps, and enhance surveillance.

## Abstract

In 2019, Indonesia and the other countries in the World Health Organization South-East Asia Region adopted the goal of measles and rubella elimination by 2023. This report describes Indonesia’s progress toward measles and rubella elimination during 2013–2022. During this period, coverage with a first dose of measles-containing vaccine (MCV) decreased from 87% to 84%, and coverage with a second MCV dose decreased from 76% to 67%. After rubella vaccine was introduced in 2017, coverage with the first dose of rubella-containing vaccine increased approximately fivefold, from 15% in 2017 to 84% in 2022. During 2013–2021, annual reported measles incidence decreased by 95%, from 33.2 to 1.4 cases per million population; reported rubella incidence decreased 89%, from 9.3 to 1.0 cases per million population. However, a large surge in measles and rubella cases occurred in 2022, with a reported measles incidence of 29 cases per million and a reported rubella incidence of 3 per million, primarily related to disruption in immunization services caused by the COVID-19 pandemic. In 2022, approximately 26 million children (an estimated 73% of the target population) received a combined measles- and rubella-containing vaccine during supplementary immunization activities completed in 32 provinces. Progress toward measles and rubella elimination in Indonesia has been made; however, continued and urgent efforts are needed to restore routine immunization services that were adversely affected by the COVID-19 pandemic and close immunity gaps to accelerate progress toward measles and rubella elimination.

## Introduction

Indonesia’s immunization program currently targets a birth cohort of approximately 4.4 million children annually. In 1982, a first dose of measles-containing vaccine (MCV1) was introduced into the routine immunization program, administered to children at age 9 months; a second MCV dose (MCV2), administered to grade 1 elementary school children (aged 7 years) was introduced in 2003. In 2013, the age of MCV2 administration was changed to 18–24 months. An MCV dose is still given to grade 1 elementary school children and recorded as the third MCV dose. Two doses of rubella-containing vaccine (RCV) were introduced into the routine immunization program in 2017 as a combined measles- and rubella-containing vaccine (MRCV). The first RCV dose (RCV1) is administered at age 9 months (as MRCV1), and the second dose (RCV2) at age 18–24 months (as MRCV2).

In 2013, Indonesia, along with the other 10 countries of the World Health Organization (WHO) South-East Asia Region (SEAR),* adopted the goal of measles elimination and control of rubella and congenital rubella syndrome (CRS), a condition that can result in miscarriage, stillbirth, or a constellation of birth defects resulting from maternal infection with rubella virus during pregnancy)^†^ by 2020 (*1*). In 2019, this goal was revised to include the elimination of both measles and rubella^§^ by 2023 (*2*). In 2021, Indonesia adopted the National Strategic Plan for Measles-Rubella Elimination 2020–2024 (*3*). The main objectives of this strategy include 1) achieving and maintaining ≥95% coverage with the first and second doses of measles- and rubella-containing vaccine (MRCV1 and MRCV2, respectively) in every district through routine immunization and supplementary immunization activities (SIAs)^¶^; 2) achieving and maintaining sensitive and timely case-based measles-rubella and CRS surveillance systems; 3) building and maintaining an accredited measles and rubella laboratory network for case confirmation that covers all 38 provinces in Indonesia; 4) ensuring preparedness and rapid response to every measles or rubella outbreak; and 5) strengthening support and partnerships. This report describes Indonesia’s progress toward measles and rubella elimination during 2013–2022.

## Methods

Administrative vaccination coverage (the number of vaccine doses administered divided by the estimated target population) is reported each year from all 7,252 districts in Indonesia to the national immunization program, where data are aggregated and reported to WHO and UNICEF through the electronic Joint Reporting Form (eJRF). WHO and UNICEF use reported administrative coverage, official estimates, and vaccination coverage survey data to generate annual estimates of national immunization coverage through routine immunization services; these estimates are used throughout this report (*4*). Measles and rubella cases are also reported to WHO and UNICEF through Indonesia’s eJRF. Genotype data are reported to the WHO measles nucleotide surveillance (MeaNS) and rubella nucleotide surveillance (RubeNS) genetic databases. This activity was reviewed by CDC, deemed research not involving human subjects, and was conducted consistent with applicable federal law and CDC policy.**

## Results

### Immunization Activities

**FIGURE 1:**
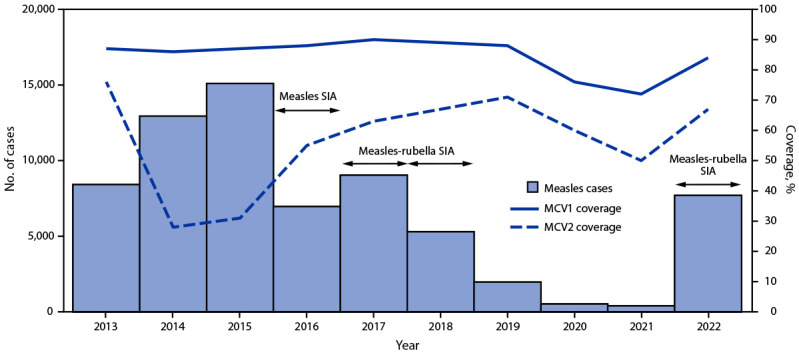
Number of reported measles cases,* estimated percentage of children who received their first and second dose of measles-containing vaccine,^†^ and supplementary immunization activities, by year^§^,^¶^,** — Indonesia, 2013–2022 **Abbreviations:** MCV1 = first dose of measles-containing vaccine in routine immunization; MCV2 = second dose of measles-containing vaccine in routine immunization; SIA = supplementary immunization activity. * Measles case data include laboratory-confirmed, epidemiologically linked, and clinically compatible cases and are reported through Indonesia’s Electronic Joint Reporting Form. ^†^ Vaccination coverage data were from World Health Organization and UNICEF estimates of national immunization coverage (https://cdn.who.int/media/docs/default-source/country-profiles/immunization/2023-country-profiles/immunization_idn_2023.pdf). ^§^ Measles SIA targeted children aged 9–59 months in 183 very high-risk districts; implemented during 2016–2017. ^¶^ Measles-rubella SIA targeted children aged 9 months to <15 years; implemented in two phases: in 2017, the SIA was conducted in six provinces in Java Island, while in 2018, the SIA was conducted in the 28 remaining provinces. ** Measles-rubella SIA in 2022 targeted children of various ages, depending on the provincial-level risk. In five provinces, the SIA targeted children aged 9 months to <15 years; in 22 provinces, it targeted children aged 9 months to <12 years; and in the remaining five provinces, the SIA targeted children aged 9–59 months.

**FIGURE 2:**
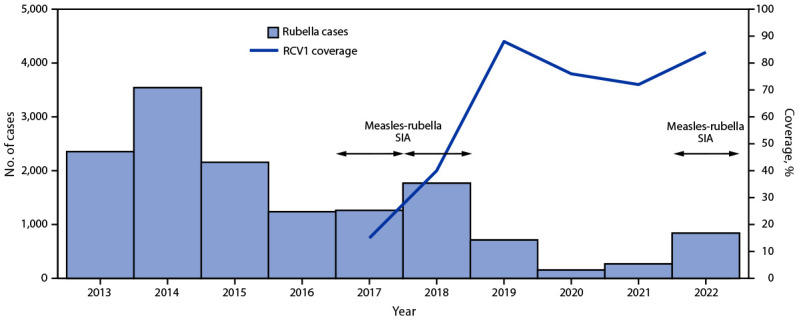
Number of reported rubella cases,* estimated percentage of children who received their first dose of rubella-containing vaccine,^†^ and supplementary immunization activities, by year^§^^,^^¶^ — Indonesia, 2013–2022 **Abbreviations:** RCV1 = first dose of rubella-containing vaccine in routine immunization; SIA = supplementary immunization activity. * Rubella case data includes laboratory-confirmed and epidemiologically linked cases and are reported through Indonesia’s Electronic Joint Reporting Form. ^†^ Vaccination coverage data were from World Health Organization and UNICEF estimates of national immunization coverage (https://immunizationdata.who.int/listing.html?topic=coverage&location=IDN); RCV1 was introduced into routine immunization in 2017. ^§^ Measles-rubella SIA targeted children aged 9 months to <15 years; implemented in two phases: in 2017, the SIA was conducted in six provinces in Java Island, while in 2018, the SIA was conducted in the 28 remaining provinces. ^¶^ Measles-rubella SIA in 2022 targeted children of various ages, depending on the provincial-level risk. In five provinces, the SIA targeted children aged 9 months to <15 years; in 22 provinces it targeted children aged 9 months to <12 years; and in the remaining five provinces, the SIA targeted children aged 9–59 months.

**Routine immunizations.** During 2013–2022, MCV1 coverage decreased approximately 3%, from 87% in 2013 to 84% in 2022; MCV2 coverage decreased approximately 12%, from 76% to 67% (Figure 1). RCV1 coverage increased from 15% in 2017 (the year RCV was introduced) to 84% in 2022 (Figure 2). During the COVID-19 pandemic, both measles and rubella vaccination coverage declined. In 2019, MCRV1 coverage was 88%; this declined to 76% in 2020 and to 72% in 2021. In 2019, MCRV2 coverage was 71%; this declined to 60% in 2020 and to 50% in 2021.

**Supplementary immunization activities.** In 2016, in response to measles outbreaks during 2014–2015, a nationwide follow-up measles SIA reached approximately 3.6 million children aged 9–59 months. In 2017 and 2018, as an integral component of RCV introduction, a MRCV SIA was conducted, reaching approximately 58 million children and adolescents aged 9 months to <15 years. In 2022, in response to the setbacks resulting from the COVID-19 pandemic, the Indonesian government sought to increase immunization coverage through Bulan Imunisasi Anak National (BIAN) or National Children Immunization Month,^ ††^ reaching approximately 26 million children with MRCV. Due in part to this campaign, MRCV1 coverage increased from 72% in 2021 to 84% in 2022, and MRCV2 coverage increased from 50% in 2021 to 67% in 2022. In addition to BIAN, other efforts implemented by Indonesian government contributed to the increase in coverage, including expanding immunization activities to include children aged <5 years and search for unimmunized or partially immunized children in remote and high-risk areas, which are defined on the basis of population immunity, surveillance quality, and the presence of vulnerable groups, such as migratory populations.

### Surveillance Activities and Measles and Rubella Incidence

Case-based measles and rubella surveillance was initiated in Indonesia in 2008^§§^; in 2019, this was transitioned to case-based fever and rash surveillance.^¶¶^ The network of WHO-accredited laboratories for measles and rubella expanded from four in 2013 to seven in 2015. During 2018–2022, the discarded nonmeasles and nonrubella rate (NMNR),*** a measure of surveillance sensitivity, approximately tripled, from 1.2 to 3.3 per 100,000 population (Table). The percentage of cases investigated within 48 hours of notification increased from 65% in 2018 to 74% in 2022. In 2022, 86% of suspected cases with adequate specimens were tested in a WHO-accredited laboratory. However, only 43% of specimens were tested within 4 days of receipt by the laboratory, and 70% of laboratory results were submitted to the immunization program within 4 days of specimen receipt, potentially delaying public health action.

**TABLE:** Reported number of measles and rubella cases, by case classification, age group, vaccination status, and surveillance indicator status — Indonesia, 2018–2022

Characteristic	Year, No. (%)				
	2018	2019	2020	2021	2022
**Measles**					
**All cases, no.**	**5,300**	**1,965**	**524**	**394**	**7,704**
Laboratory-confirmed*	861 (16)	639 (33)	310 (50)	132 (34)	4,844 (63)
Epidemiologically linked^†^	153 (3)	22 (1)	0 (0)	1 (0)	103 (1)
Clinically compatible^§^	4,286 (81)	1,304 (66)	214 (50)	261 (66)	2,757 (36)
Incidence^¶^	19.8	7.3	1.9	1.4	28.8
Measles genotypes (no.)	D8 (5)	—	—	—	D8 (54), B3 (47)
**Age group of patients with laboratory-confirmed and epidemiologically linked measles**					
<9 mos	64 (6)	20 (3)	11 (4)	7 (5)	484 (10)
9 mos–4 yrs	131 (13)	43 (6)	24 (8)	13 (10)	356 (7)
5–9 yrs	151 (15)	204 (31)	78 (25)	19 (14)	1,539 (31)
10–14 yrs	112 (11)	40 (6)	29 (9)	11 (8)	358 (7)
≥15 yrs	150 (15)	64 (10)	32 (10)	22 (17)	307 (7)
Unknown or missing	406 (40)	290 (44)	136 (44)	61 (46)	1,903 (38)
**MCV doses received by patients with laboratory-confirmed or epidemiologically linked measles**					
≥2	0 (—)	25 (4)	11 (5)	20 (15)	290 (6)
1	1 (—)	43 (6)	24 (8)	12 (9)	267 (5)
0	53 (5)	85 (13)	93 (30)	45 (34)	3,178 (64)
Unknown	960 (95)	508 (77)	182 (59)	56 (42)	1,212 (24)
**Rubella**					
**All cases, no.**	**1,767**	**713**	**155**	**268**	**839**
Laboratory-confirmed**	1,767 (100)	710 (100)	155 (100)	267 (100)	839 (100)
Epidemiologically linked^††^	0 (—)	3 (—)	0	1 (—)	0 (—)
Incidence^¶^	6.60	2.60	0.60	0.98	3.05
Rubella genotypes	NA	NA	NA	NA	NA
**Age group of patients with laboratory-confirmed and epidemiologically linked rubella**					
<9 mos	17 (1)	12 (2)	6 (4)	8 (3)	30 (4)
9 mos–4 yrs	48 (3)	73 (10)	25 (16)	35 (13)	98 (12)
5–9 yrs	364 (21)	93 (13)	25 (16)	39 (15)	151 (18)
10–14 yrs	347 (20)	66 (9)	13 (8)	25 (9)	68 (8)
≥15 yrs	388 (22)	76 (11)	22 (14)	28 (10)	133 (16)
Unknown or missing	603 (34)	393 (55)	64 (41)	133 (50)	359 (48)
**RCV doses received by patients with laboratory-confirmed or epidemiologically linked rubella**					
≥2	7 (0)	35 (5)	15 (10)	40 (15)	130 (15)
1	22 (1)	68 (10)	20 (13)	35 (13)	101 (12)
0	72 (4)	75 (11)	40 (26)	88 (33)	348 (41)
Unknown	1,666 (94)	535 (75)	80 (52)	105 (39)	260 (31)
**Congenital rubella syndrome**					
**All suspected cases, no.**	**275**	**664**	**457**	**916**	**1,026**
Laboratory-confirmed^§§^	89 (32)	35 (5)	10 (2)	29 (3)	25 (2)
Clinically compatible^¶¶^	99 (36)	176 (27)	100 (22)	200 (22)	148 (14)
Discarded***	87 (32)	453 (68)	347 (76)	687 (75)	853 (83)
**Surveillance and program implementation**					
Provinces with case-based fever and rash surveillance^†††^	34 (100)	34 (100)	34 (100)	34 (100)	34 (100)
WHO-accredited measles and rubella laboratories, no.	7	7	7	7	7
Provinces completing measles-rubella SIA	28 (80)	0 (—)	0 (—)	0 (—)	34 (100)
Surveillance performance indicators					
No. of discarded NMNR cases^§§§^	3,065	5,099	2,188	2,269	9,149
No. of discarded NMNR cases per 100,000 population, national level (target: ≥2)	1.2	1.0	0.8	0.8	3.3
Districts with NMNR discard rate ≥2	71 (14)	123 (24)	42 (8)	73 (14)	230 (45)
% of suspected cases adequately investigated ≤48 hrs of notification (target: ≥80)	65	62	60	71	74
% of suspected cases with adequate specimens^¶¶¶^ tested for measles and rubella in a proficient laboratory**** (target: ≥80)	96	94	91	92	86
% of samples tested ≤4 days of specimen receipt in laboratory (target: ≥80) ^††††^	86	66	64	84	43
% of results received by program ≤4 days of specimen receipt (target: ≥80) ^§§§§^	83	63	57	83	70
% of surveillance units reporting weekly to national level on time (target: ≥80)	47	40	77	70	89

**Abbreviations:** IgM = immunoglobulin M; MCV = measles-containing vaccine; NA = not available; NMNR = nonmeasles, nonrubella; RCV = rubella-containing vaccine; SIA = supplementary immunization activity; WHO = World Health Organization.

* Defined as a case that meets the suspected case definition and is laboratory-confirmed (serologically or virologically) as measles.

^†^ Defined as a case that meets the suspected case definition, which is found as part of a laboratory-confirmed measles outbreak investigation but does not have a laboratory specimen collected.

^§^ Defined as a case that meets the suspected case definition without an adequate laboratory specimen collected and without epidemiological linkage to another laboratory-confirmed communicable disease.

^¶^ Cases per 1 million population.

** Defined as a case that meets the suspected case definition and is laboratory-confirmed as rubella.

^††^ Defined as a case that meets the suspected case definition, which is found as part of a laboratory-confirmed rubella outbreak investigation but does not have a laboratory specimen collected.

^§§^ Defined as a case that meets the suspected case definition and is laboratory-confirmed as congenital rubella syndrome.

^¶¶^ Defined as a case that meets the suspected case definition and the clinically compatible case definition but does not have an adequate laboratory specimen collected.

*** Defined as a case that meets the suspected case definition but does not meet the clinically compatible case definition or has negative laboratory testing for congenital rubella syndrome.

^†††^ Case-based fever and rash surveillance identifies suspected cases as illness in any person with fever and maculopapular (nonvesicular) rash or in any person in whom a clinician suspects measles or rubella infection.

^§§§^ Suspected cases that have been investigated and discarded as not measles or rubella by 1) laboratory result negative for measles and rubella through serum sample testing in a proficient laboratory and 2) no epidemiological linkage to another measles or rubella case.

^¶¶¶^ Serum specimen collected ≤28 days (for serology) and throat or urine samples collected ≤7 days (for virology) after rash onset.

**** A WHO-accredited laboratory that has an established quality assurance program or one with oversight by a WHO-accredited laboratory.

^††††^ Samples tested for measles and rubella IgM ≤4 days after sample receipt by laboratory.

^§§§§^ Laboratory results for measles and rubella IgM received by program ≤4 days after sample receipt by laboratory.

Sentinel CRS surveillance was initiated in 13 hospitals in Indonesia in 2015 and was expanded to 22 hospitals in 2022. During 2018–2022, the national reporting rate for suspected CRS cases (a marker of CRS surveillance sensitivity) increased 85% from 1.71 to 3.16 per 10,000 live births. In 2018, among 275 suspected CRS cases, 89 (32%) were laboratory-confirmed, 99 (36%) were clinically confirmed as CRS, and 87 (32%) were discarded. In 2022, among 1,026 suspected CRS cases, 25 (2%) were laboratory-confirmed, 148 (14%) were clinically confirmed, and 853 (83%) were discarded. Despite the increase in the number of suspected cases, likely related to increased detection through increased surveillance sites, the percentage of laboratory or clinically confirmed CRS cases decreased, likely representing a decline in CRS incidence following introduction of RCV.

During 2013–2022, measles incidence decreased from 33.2 cases per million population to a low of 1.4 in 2021 but sharply increased to 28.8 in 2022 (Table). In 2022, 88% of patients with laboratory-confirmed or epidemiologically linked measles had received no MCV doses or had an unknown vaccination history. After the introduction of RCV and a wide age-range MRCV SIA, rubella incidence declined from 9.3 cases per million in 2013 to a low of 0.6 in 2020 but increased to 3.1 in 2022. Similar to what was observed with measles surveillance, 72% of persons with laboratory-confirmed or epidemiologically linked rubella had received no RCV doses or had unknown vaccination history.

Measles virus genotypes detected and reported included D8 in 2018 and 2022 and B3 in 2022. No rubella virus genotypes were detected or reported (Table).

## Discussion

During 2013–2022, Indonesia implemented substantial efforts toward measles and rubella elimination, including introducing RCV and conducting a wide age-range MRCV SIA. During 2013–2019, MCV1 coverage was stable (86%–90%); MCV2 coverage declined during 2014–2015, likely because of a change in age of administration in 2013, followed by an increase in coverage during 2016–2019. After introduction of RCV in 2017, RCV1 coverage increased steadily through 2019. However, the COVID-19 pandemic resulted in substantial declines in both coverage with MCV1, MCV2, and RCV1 and in sensitivity of measles and rubella (MR) surveillance during 2020–2021, because of temporary closing of immunization posts, movement restrictions, and repurposing of immunization and surveillance staff to COVID-19 activities. Similar COVID-19 pandemic-related setbacks were seen in other WHO SEAR countries (*5**,**6*).

Existing immunity gaps, widened by immunization coverage setbacks during the COVID-19 pandemic, led to substantial increases in the number of measles and rubella cases in 2022 compared with 2021. This is consistent with an independent review of progress toward measles and rubella elimination in WHO SEAR during October–November 2021, which concluded that Indonesia would not achieve measles and rubella elimination by 2023 (*7*).

In 2022, recovery efforts were accelerated with BIAN immunization activities. MRCV1 and MRCV2 coverage rates have rebounded to levels approaching those achieved in 2019. Despite this improvement, to accelerate progress toward elimination, Indonesia will need to continue to intensify efforts to enhance MR surveillance and close immunity gaps among eligible children through SIAs, with a particular focus in districts with low coverage (*8*). 

### Limitations

The findings in this report are subject to at least three limitations. First, coverage estimates based on administrative data might be inaccurate because of errors in recording doses administered or in estimating the target population. Second, surveillance data might underestimate actual disease incidence because surveillance sensitivity was low: children who had measles or rubella might not have been brought in for care, not all cases in patients who sought care might have received a proper diagnosis, and some diagnosed cases might not have been reported. Finally, few specimens were submitted for sequencing, so genotype data might not reflect predominant circulating genotypes.

### Implications for Public Health Practice

COVID-19 caused substantial setbacks to Indonesia’s MR elimination program. In 2022, intensified efforts to increase immunization coverage and improve MR surveillance sensitivity resulted in program recovery. Indonesia’s large annual birth cohort represents an important opportunity to prevent illness and death from measles and rubella viruses. Urgent actions are still needed to accelerate progress toward MR elimination, including vaccinating all eligible children and optimizing surveillance sensitivity.

## Footnotes

* Bangladesh, Bhutan, Burma, India, Indonesia, Maldives, Nepal, North Korea, Sri Lanka, Thailand, and Timor-Leste.

† Measles elimination is defined as the absence of endemic measles cases for ≥12 months in the presence of adequate surveillance. Rubella and congenital rubella syndrome control is defined as a 95% reduction in disease incidence from the 2013 level.

§ Rubella elimination is defined as the absence of endemic rubella cases for ≥12 months in the presence of adequate surveillance.

¶ In Indonesia, follow-up SIAs for measles and rubella conducted during 2013–2022 were carried out using two target age ranges. SIAs focused on all children and adolescents aged 9 months to <15 years and were conducted with the goal of eliminating susceptibility to measles and rubella in the general population. Other SIAs focused on children aged 9–59 months with the goal of eliminating any measles and rubella susceptibility that had accumulated in recent birth cohorts and of protecting the estimated 2%–5% of children who did not respond to the first measles vaccine dose.

** 45 C.F.R. part 46; 21 C.F.R. part 56; 42 U.S.C. Sect. 241(d), 5 U.S.C. Sect. 552a, 44 U.S.C. Sect. 3501 et seq.

†† BIAN or National Children Immunization Month efforts included immunization activities (selectively giving ≥1 dose of measles-rubella containing vaccine to children aged 12–59 months who missed or had not received the appropriate number of measles and rubella (MR) doses) and an MR SIA targeting all children aged 9–59 months, regardless of previous vaccination status. BIAN was implemented in 32 of 34 provinces in Indonesia.

§§ For case-based measles and rubella surveillance, a suspected case was defined as fever and maculopapular rash with cough, coryza, or conjunctivitis, or any illness in a person that a clinician or health worker suspects to be measles infection.

¶¶ For case-based acute fever and rash surveillance, a suspected case was defined as fever and maculopapular rash, or any illness in a person that a clinician or health worker suspects to be measles or rubella infection.

*** A discarded case is defined as a suspected case that has been investigated and determined to be neither measles nor rubella by 1) laboratory testing in a proficient laboratory or 2) epidemiologic investigation with no linkage to another confirmed measles or rubella case.
