# *Erratum:* Vol. 72, No. 42

**DOI:** 10.15585/mmwr.mm7244a4

**Published:** 2023-11-03

In the report, “Progress Toward Measles and Rubella Elimination — Indonesia, 2013–2022,” on page 1135, the last sentence of the Methods section should have read, “This activity was reviewed by CDC, **deemed not research,** and was conducted consistent with applicable federal law and CDC policy.**” The corresponding footnote should have read, “45 C.F.R. part **46.102(l)(2), **21 C.F.R. part 56; 42 U.S.C. Sect. 241(d); 5 U.S.C. Sect. 552a; 44 U.S.C. Sect. 3501 et seq.”

